# Sphingosine 1-Phosphate in Acute Dengue Infection

**DOI:** 10.1371/journal.pone.0113394

**Published:** 2014-11-19

**Authors:** Laksiri Gomes, Samitha Fernando, Randika Heshan Fernando, Nilanka Wickramasinghe, Narangoda Liyanage Ajantha Shyamali, Graham S. Ogg, Gathsaurie Neelika Malavige

**Affiliations:** 1 Centre for Dengue Research, Department of Microbiology, Faculty of Medical Sciences, University of Sri Jayawardanapura, Gangodawila, Sri Lanka; 2 MRC Human Immunology Unit, Weatherall Institute of Molecular Medicine, Oxford NIHR Biomedical Research Centre and University of Oxford, Oxford, United Kingdom; 3 Department of Dermatology, Churchill Hospital, Oxford, United Kingdom; Agency for Science, Technology and Research - Singapore Immunology Network, Singapore

## Abstract

**Background:**

Vascular leak is the hallmark of severe dengue infections and leads to complications such as shock and multi-organ failure. Although many mediators have been implicated in the vascular leak in dengue, the role of sphingosine 1-phosphate (S1P) has not been investigated.

**Metholodology/Principal findings:**

As S1P has been shown to be important in barrier integrity, we assessed the S1P levels in 28 patients with acute dengue and 12 healthy individuals. The S1P levels were significantly lower in patients with acute dengue (p = 0.002) and the levels in patients with grade IV dengue haemorrhagic fever (DHF) were significantly lower than those with dengue fever (p = 0.005). We then investigated the kinetics of S1P levels throughout the course of the illness in another 32 patients in serum samples obtained twice a day. We found that S1P levels were low throughout the course of illness and S1P levels were <0.5 µM in 12/23 patients with DHF when compared to 1/9 with DF.

**Conclusions/Significance:**

As S1P has shown to be important in the endothelial barrier integrity and increases transendothelial resistance, low levels of S1P in acute dengue infection are likely to contribute to increased vascular permeability.

## Introduction

Dengue infections are one of the most important vector borne diseases and are estimated to infect 390 million individuals annually [1]. The incidence of dengue has dramatically increased in many affected countries causing a significant disease burden [2]. Currently there is no licensed vaccine for its prevention, or an effective antiviral for its treatment. Management of acute dengue consists of early detection of complication and meticulous fluid management [3].

Dengue infection is caused by one of the four dengue virus (DV) serotypes which are closely related. Although the majority of individuals who are infected with the DV develop asymptomatic infection or an undifferentiated fever, some develop dengue fever (DF) or more severe forms of clinical disease such as dengue hemorrhagic fever (DHF), dengue shock syndrome or expanded syndrome of dengue infection [3]. The initial phase of infection is characterized by a high fever (febrile phase) and coincides with the viraemia [4]. The patient may then progress to the critical phase during day 3 to 7 of illness in which there is increased vascular permeability leading to fluid leakage and shock [4], [5]. Fluid leakage is the hallmark of severe dengue infection and this results in complications such as pleural effusions, ascites and if left untreated, shock and multi-organ failure [3]. The critical phase usually lasts for 24 to 48 hours and then the patient progressed to the recovery phase where the fluid is reabsorbed and the patient typically improves. Although infection of endothelial cell lines in vitro has been shown [6]–[8], due the relatively short duration of vascular leak phase in acute dengue infection, many believe that the increased vascular permeability is due to endothelial dysfunction or activation rather than damage associated with endothelial infection [9]. Serum from patients with DHF has been shown to alter the vascular permeability and it has been suggested that a cytokine or a mediator present in serum is the likely cause of increase in vascular permeability [10]. Although many mediators have been implicated [9], [11], [12], the role of vasoactive mediators such as sphingosine 1-phosphate in endothelial barrier integrity in dengue has not been investigated.

S1P is a signaling phospholipid that is predominantly released from platelets and rapidly enhances transendothelial resistance [13], [14]. S1P is known to involved in lymphocyte egress, and especially T cell egress from lymph nodes and Peyers Patches to blood [15]. Low S1P levels have been found in diseases associated with vascular leak such as cerebral malaria [16]. In influenza and malaria murine models, use of S1P analogues has reduced microvascular leak and improved the outcome [13], [17], [18]. Use of S1P analogues has also been shown to reduce immunopathology, improved survival and reduction of the production of inflammatory cytokines without increasing or prolonging the duration of viraemia [19]. Moreover, S1P analogues have also shown to reduce activation of monocytes, NK cells and inhibit antigen specific T cell responses in murine models [19]. S1P has also been shown to counteract the effects of other mediators that cause increase vascular permeability such as vascular endotheial growth factor (VEGF) on the endothelial by blocking VE-cadherin internalization in response to VEGF and thereby improving endothelial integrity [13]. High levels of VEGF has been have been found in dengue and shown to associate with vascular leak [12]. Therefore, S1P could play a significant role in preserving the endothelial barrier integrity in acute dengue infection and also have many immunomodulatory effects. In this study we have shown that S1P are significantly lower in patients with acute dengue infection which could result in increased vascular permeability. Therefore, the use of S1P analogues could have a potential benefit in the treatment of acute dengue infection.

## Materials and Methods

In order to determine if S1P levels were lower in patients with acute dengue we first recruited a cohort of 28 adult patients with clinical features suggestive of dengue infection who were admitted to a general medical ward in a tertiary care hospitals in Colombo during the year 2013. Blood samples were obtained during day 6 and 7 day of illness. The study was approved by the Ethics Review Committee of the University of Sri Jayawardanapura and patients were recruited following informed written consent. 8 of these patients had DHF and 20 had DF. 12 healthy dengue seropositive individuals were also recruited for determining S1P levels in healthy individuals.

In order to determine the kinetics of S1P levels throughout the course of the illness we recruited an additional 32 adult patients. The onset of illness was defined as the time of onset of fever. If the patient was recruited following 3 days of fever, it was considered that the duration of illness was 72 hours. Serial blood samples were taken in the morning (6 a.m.) and again at 1.00p.m., from the time of admission to the time of discharge from hospital in this cohort of 32 patients. The initial blood sample was taken between 72–120 hours of illness (mean 106.2 hours, SD±19.2), which was when the patients were first admitted to hospital. All clinical features such as presence of fever, abdominal pain, vomiting, bleeding manifestations, hepatomegaly, blood pressure, pulse pressure and evidence of fluid leakage were recorded several times each day. The time of onset of fever was considered as 0 hours and the duration of illness was considered from the onset of fever. The full blood counts and the alanine transaminase (ALT) and aspartate transaminase (ALT) levels were done during the course of the illness. The severity of dengue infection was classified according to the 2011 WHO dengue diagnostic criteria [3]. Accordingly, patients with a rise in haematocrit above≥20% of the baseline haematocrit or clinical or ultrasound scan evidence of plasma leakage in a patient was classified as having DHF. Shock was defined as having cold clammy skin, along with a narrowing of pulse pressure of ≤20 mmHg. According to this definition 22 had DHF and 10 DF. Of the 22 patients who were classified as having DHF, 3 had grade III DHF as they developed shock, one had grade II DHF and 18 had grade I DHF according to the 2011 WHO dengue guidelines [3]. Of the 10 patients who had DF, although some patients were quite ill with very low platelet counts <25,000 cells/mm^3^, and high liver enzymes, they were classified has having DF as there was no evidence of fluid leakage according to the WHO guidelines [3].

### Quantitative of serum S1P levels

Quantitative S1P assays were done in duplicate on all serum samples according to manufacturer's instructions (Echelon Biosciences, Inc., USA). The levels were expressed as µM. In the 28 patients who were initially recruited to determine if S1P levels were low in acute dengue, the levels were measured only at one time point from blood obtained at the critical phase. In the cohort 32 patients who were subsequently recruited to determine the kinetics of S1P, the levels were done twice a day from time of recruitment, to time of discharge from the hospital.

### Confirmation of dengue infection

Acute dengue infection was confirmed in the serum samples using the NS1 early dengue ELISA (Panbio, Australia) or with the commercial capture-IgM and IgG enzyme-linked immunosorbent assay (ELISA) (Panbio, Brisbane, Australia). The ELISA was performed and the results were interpreted according to the manufacturer's instructions. This ELISA assay has been validated as both sensitive and specific for primary and secondary dengue virus infections [20], [21].

### Statistical analysis

Statistical analysis was performed using Graph pad PRISM version 6. As the data were not normally distributed, differences in means were compared using the Mann-Whitney U test (two tailed). Kruskal-Wallis test was used to determine the differences in S1P levels in healthy individuals, patients with DF and DHF. Degree of association between serum S1P and other clinical parameters were analysed using Spearmans correlation.

## Results

As reduction of S1P levels has been shown to be associated with plasma leakage in other disease conditions [16], we initially investigated the S1P levels in a cohort of patients with acute dengue infection and healthy individuals. 8 of these patients had grade IV DHF (dengue shock syndrome) based on the 2011 WHO disease classification [3] and 20 had DF. 3 of the 8 patients with DHF succumbed to their illness and the samples were collected approximately 12–24 hours before death. The other 5/8 DHF develop shock. The clinical data of these 28 patients and the 32 patients we later did serial blood sampling is shown in table 1.

**Table 1:** Clinical and laboratory characteristics of the 60 patients recruited.

Clinical characteristic	DHF N = 30	DF N = 30
Pleural effusions or ascites	18 (60)	0
Shock	11 (36.7)	0
Rise in Haematocrit >20% of baseline	27 (90)	0
Bleeding manifestations	5 (16.6)	2 (6.7)
AST		
<200 IU	12 (40)	20 (66.7)
200–500 IU	11 (36.7)	7 (23.3)
500–999 IU	4 (13.3)	2 (6.7)
>1000 IU	3 (10)	1 (3.3)
ALT		
<200 IU	14 (46.7)	24 (80)
200–500 IU	11 (36.7)	5 (16.7)
500–999 IU	3 (10)	1 (3.3)
>1000 IU	2 (6.7)	0 (0)
Lowest platelet counts (cells/mm^3^)		
<25,000	16 (53.3)	7 (23.3)
25,000–50,000	10 (33.3)	9 (30)
50,000–99,999	4 (13.3)	12 (40)
>100,000	0 (0)	2 (6.7)

**Figure 1:**
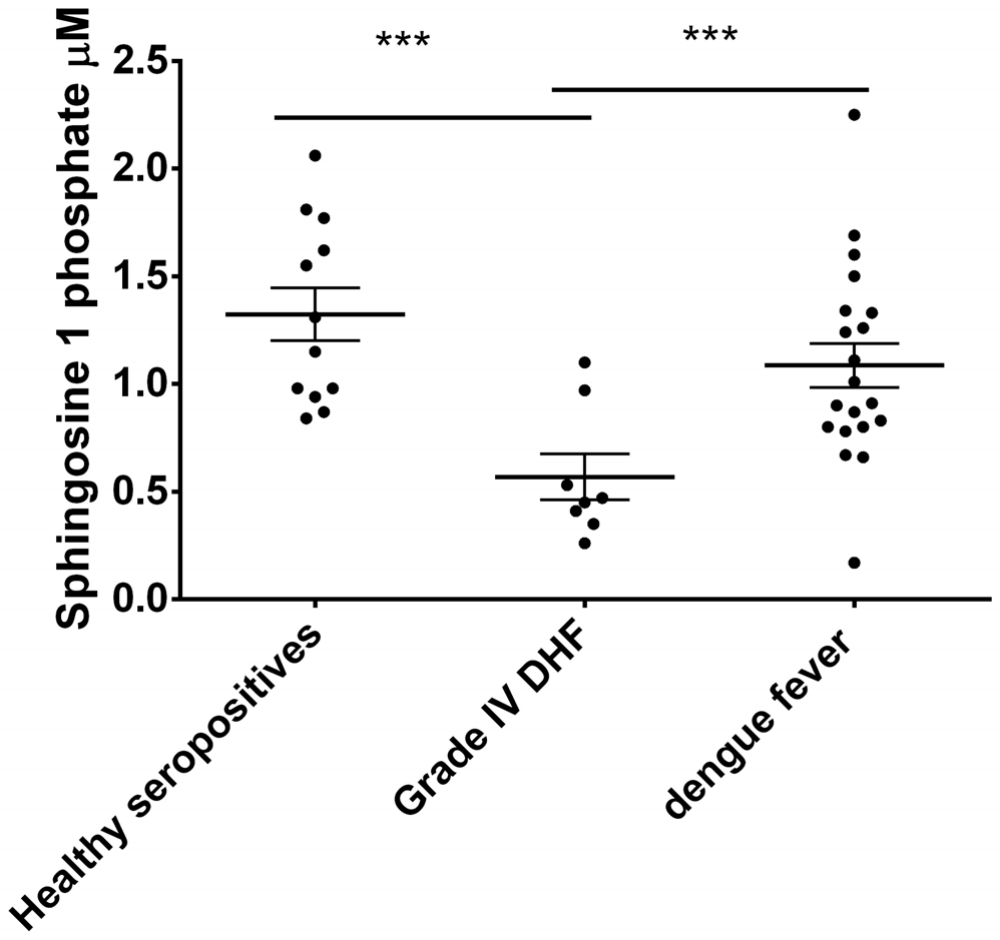
S1P levels in patients with grade IV DHF, patients with DF and healthy individuals. Serum S1P was measured by quantitative ELISA in individuals with grade IV DHF (n = 8), DF (n = 20) and healthy controls (n = 12). The lines represent the mean and the error bars represent the standard error of mean. ***p = 0.005.

In the 28 patients we initially recruited, we found that S1P levels were significantly lower in patients with acute dengue infection (p = 0.002) when compared to healthy individuals. The S1P levels were significantly lower (p = 0.005) in those with grade IV DHF (median 0.46, IQR 0.36 to 0.86 µM) when compared to those with DF (median 0.96, IQR 0.8 to1.34 µM) (Fig 1).

### Kinetics of S1P levels in patients with DHF and DF

Since we found that S1P levels were significantly lower in patients with severe forms of dengue, S1P levels were also analyzed in serial samples in 32 patients with DHF and DF to determine if changes in S1P levels coincided with the phase of vascular leak in acute dengue infection. According 2011 WHO disease classification [3], 22 had DHF and 10 DF. The clinical characteristics of these 32 patients are shown in Table 2.

**Table 2:** Clinical and laboratory characteristics of the patients in whom serial S1P levels were determined.

Patient number	Pleural effusions/ascites	Rise in Haematocrit >20% of baseline	Lowest platelet counts	Shock	Bleeding manifestations	NS1 antigen positivity on admission	DF/DHF and grade
1	No	Yes	32,000	No	No	yes	DHF1
2	No	Yes	42,000	No	No	yes	DHF1
3	Yes	Yes	19,000	No	No	yes	DHF1
4	Yes	Yes	58,000	Yes	Melaena	yes	DHF3
5	Yes	Yes	43,000	No	No	yes	DHF1
6	No	Yes	45,000	No	No	yes	DHF1
7	No	Yes	42,000	No	No	yes	DHF1
8	No	Yes	56,000	No	No	yes	DHF1
9	No	No	27,000	No	No	yes	DF
10	No	No	112,000	No	No	no	DF
11	No	No	91,000	No	No	no	DF
12	No	No	86,000	No	No	no	DF
13	Yes	No	10,000	No	No	no	DHF1
14	Yes	Yes	10,000	No	No	no	DHF1
15	Yes	No	16,000	No	No	no	DHF1
16	Yes	Yes	10,000	Yes	No	no	DHF3
17	Yes	Yes	18,000	Yes	No	yes	DHF3
18	No	Yes	44,000	No	No	yes	DHF1
19	No	Yes	69,000	No	Gum bleeding	yes	DHF2
20	No	Yes	42,000	No	No	yes	DHF1
21	Yes	No	16,000	No	No	no	DHF1
22	No	Yes	86,000	No	No	yes	DHF1
23	No	No	82,000	No	No	yes	DF
24	No	Yes	6,000	No	No	no	DHF1
25	No	No	24,000	No	No	no	DF
26	No	Yes	12,000	No	No	yes	DHF1
27	No	No	84,000	No	No	no	DF
28	No	No	90,000	No	No	yes	DF
29	No	No	82,000	No	No	yes	DF
30	No	Yes	12,000	No	No	yes	DHF1
31	No	No	35,000	No	No	yes	DF
32	Yes	Yes	12,000	No	No	yes	DHF1

DHF1: Grade I DHF, DHF2: grade II DHF, DHF3: grade III DHF.

**Figure 2:**
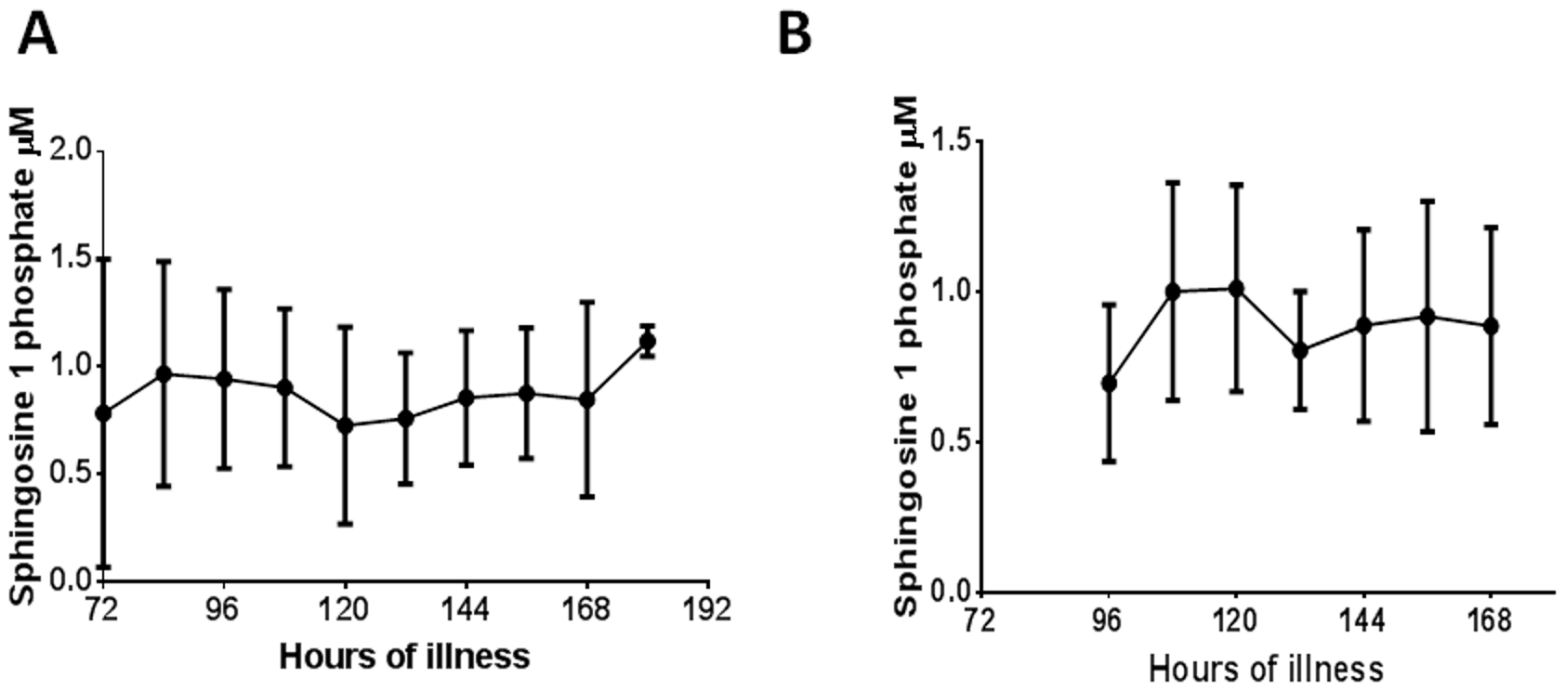
Variation of serum S1P levels throughout the course of the illness in patients with DF and DHF. A: S1P levels in patients with DHF (n = 22). The bars indicate the mean and the standard error of the mean. The time of onset of fever was considered as 0 hours and the number of hours of illness considered from the time of onset of fever. B: S1P levels in patients with DF(n = 10). The bars indicate the mean and the standard error of the mean. The time of onset of fever was considered as 0 hours and the number of hours of illness considered from the time of onset of fever.

**Figure 3:**
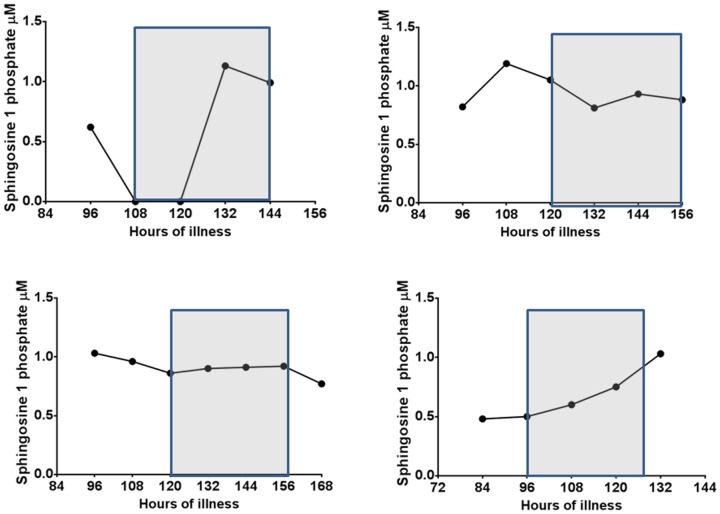
Variation of serum S1P levels in patients with DHF: Kinetics of S1P levels in 4 patients with DHF. The critical phase is shaded.

**Figure 4:**
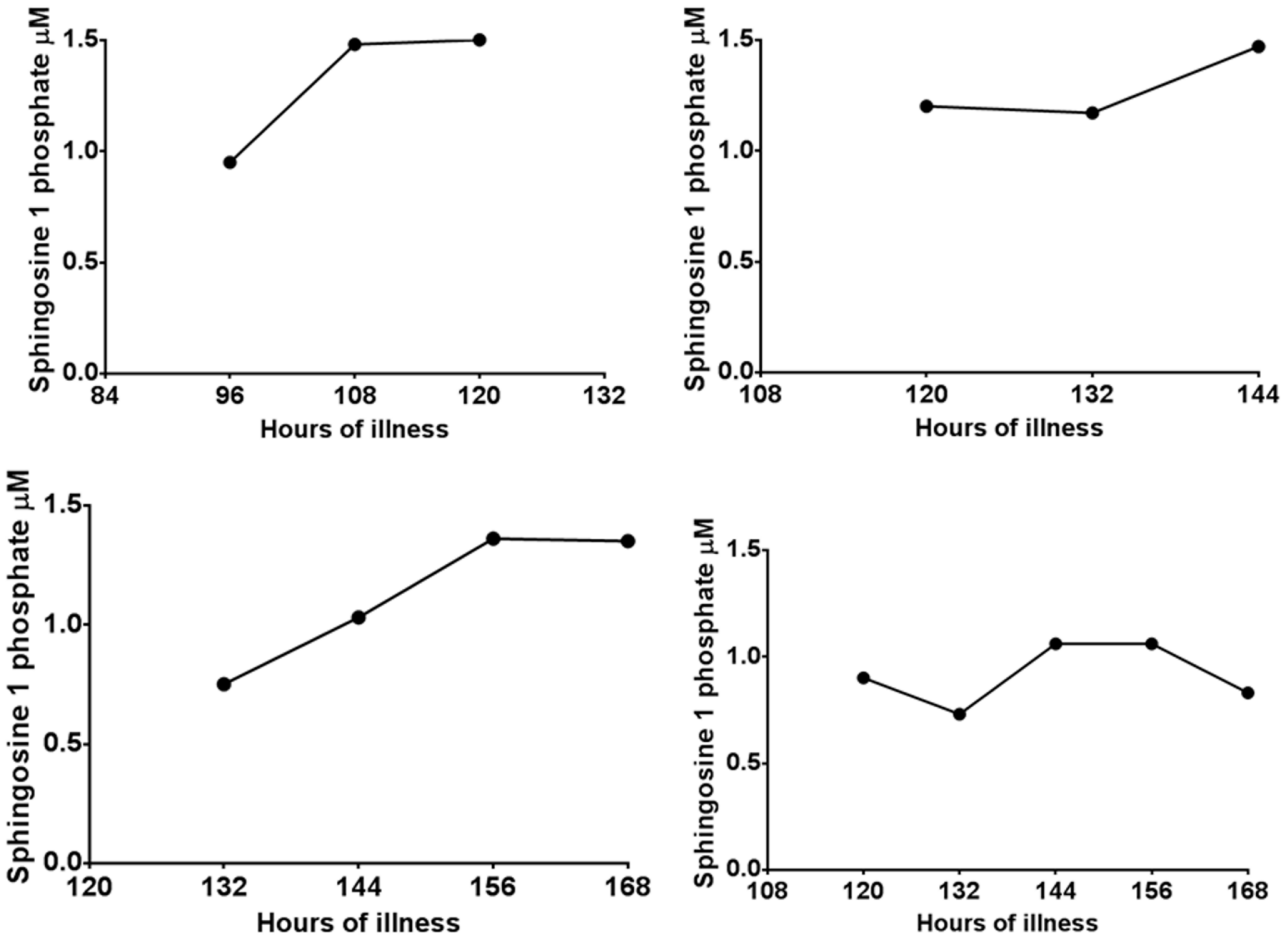
Variation of serum S1P levels in patients with DF: Kinetics of S1P levels in 4 patients with DF.

Although the majority of patients with DHF had lower S1P levels especially in the critical phase (Fig 2A), some patients with DF also had quite low levels at certain time points during the course of the illness (Fig 2B). However overall 12/22 (54.5%) of those with DHF and 1/10 (10%) of patients with DF had values <0.5 µM at sometime point during the course of the illness. The levels of S1P along with the critical phase are shown in 4 patients with DHF (Fig. 3) and in 4 patients with DF (Fig 4). Since patients with DF stayed in hospital for a shorter duration that patients with DHF, only 3 to 4 time points could be obtained whereas 5 to 7 time points could be obtained from patients with DHF.

### Association of S1P levels with laboratory parameters

**Figure 5:**
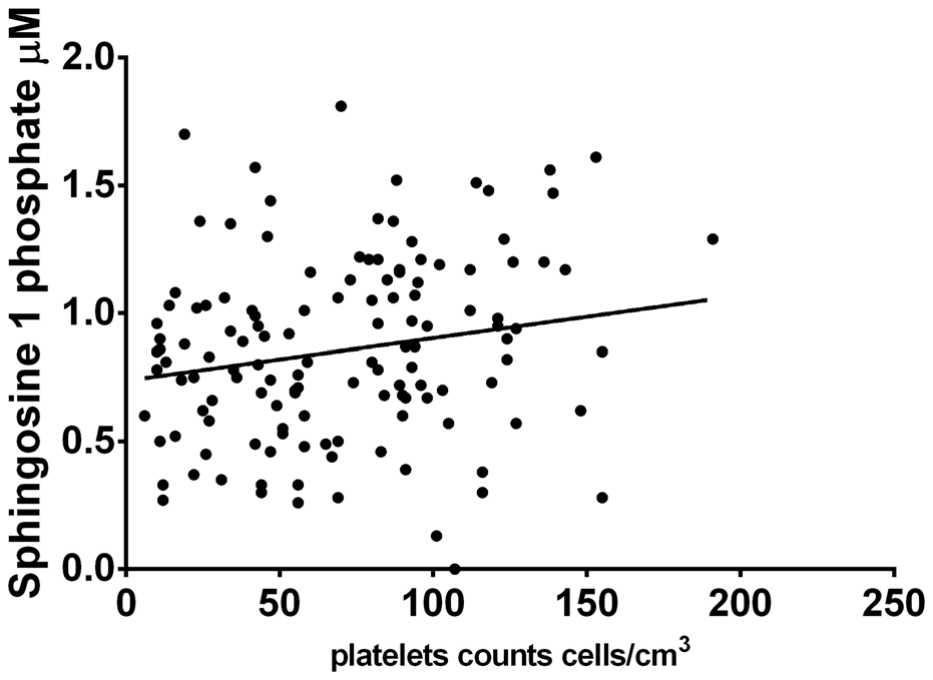
Correlation of serial serum S1P levels with serial platelet counts in patients with DF (n = 10) and DHF (n = 22). Spearmans r = 0.18, p = 0.04.

The main source of S1P is thought to be platelets. In the 32 patients in whom S1P levels were measured serially, we went on to determine if the serial S1P levels correlated with serial platelet counts obtained throughout the course of the illness in these patients. The serial S1P levels in patients with both DF and DHF (n = 32), significantly correlated with the serial platelet counts (Spearmans r = 0.18, p = 0.04) (Fig 5). S1P is also shown to be involved in lymphocyte egress, and indeed T cell egress from lymph nodes and Peyers Patches to blood is controlled by S1P [15]. However, we did not find any association between total lymphocyte counts in patients with acute dengue infection and S1P levels, probably as one of the main causes of lymphopenia in acute dengue appears to be due to T cell apoptosis [22], [23].

## Discussion

In this study we have found that S1P levels were significantly lower in patients with more severe forms of dengue infection and the levels remain low throughout the course of the illness. Although patients with both DF and DHF had quite low levels throughout the course of the illness, 12/22 patients with DHF had levels <0.5 µM at sometime point in the illness, whereas such low levels were only seen in 1/10 patients with DF. S1P has been shown to be important in the endothelial barrier integrity and increases transendothelial resistance [13], [14]. Therefore, it is likely that low S1P levels in acute dengue infection could be contributing to the microvascular leak in acute dengue infection. Although S1P analogues have not been used in humans in diseases associated with increase in vascular permeability such as in sepsis, in rat models of sepsis, they have been shown to significantly reduce loss of plasma [24]. Other mediators such as VEGF also contribute to vascular leak in dengue [12]. S1P inhibits the effects of VEGF on the vascular endothelium and S1P analogues inhibit VEGF induced vascular leak in mice models [25]. Therefore, it is speculated that S1P analogues such as fingolimod could potentially have an effect of reducing vascular leak in acute dengue infection and thus preventing complications associated with vascular leak such as pleural effusions, ascites, shock and multi organ failure.

Platelets are believed to be one of the main sources of S1P [14] and serum S1P levels correlated significantly with the platelet counts in patients with acute dengue. Profound thrombocytopenia is seen in patients with acute dengue and platelet counts are significantly lower in those who develop more severe forms of dengue [26], [27]. Since platelets are the main source of S1P, thrombocytopenia as likely to cause low S1P levels. However, prophylactic platelet transfusions have not shown to be of any benefit in the management of dengue [28] and due to the potential risks and lack of benefit, this is currently not recommended [3].

S1P has shown to have many immunomodulatory functions and S1P analogues have been shown to reduce production of inflammatory cytokines and inhibit the activation of antigen specific T cells without affecting virus clearance in murine models and ferrets infected with influenza [17], [18], [29]. S1P receptor agonists improve survival in other infections associated with a cytokine storm such as respiratory syncytial virus infection [30]. However, clinical trial data of the use of S1P analogue Fingolimod has shown that patients who are on this drug had a higher frequency of viral infections [31]. In murine models of experimental autoimmune encephalomyelitis (EAE) although Fingolimod reduced EAE, it reduced survival following an influenza virus challenge as it inhibited antigen specific T cell activation and cytotoxic function [31]. The role of T cells in the pathogenesis of acute dengue is debated [5] although proinflammatory cytokines have also been shown to play a significant role in the pathogenesis of acute dengue infection [32], [33]. Therefore, the use of a S1P analogue in the treatment of acute dengue could be potentially beneficial and warrants investigation using murine models.

In summary, S1P levels are significantly lower in patients with more severe forms of dengue and remain low throughout the course of illness. Since S1P is known to preserve endothelial barrier integrity, the potential role of S1P analogues in the treatment of microvascular leakage in acute dengue infections should be further evaluated.
